# Laser-Induced Breakdown Spectroscopy vs. Fluorescence Spectroscopy for Olive Oil Authentication

**DOI:** 10.3390/foods14061045

**Published:** 2025-03-19

**Authors:** Marios Bekogianni, Theodoros Stamatoukos, Eleni Nanou, Stelios Couris

**Affiliations:** 1 Department of Physics, University of Patras, 26504 Patras, Greece; up1068704@ac.upatras.gr (M.B.); up1071016@ac.upatras.gr (T.S.); e.nanou@iceht.forth.gr (E.N.); 2 Institute of Chemical Engineering Sciences (ICE-HT), Foundation for Research and Technology-Hellas (FORTH), 26504 Patras, Greece

**Keywords:** laser-induced breakdown spectroscopy—LIBS, fluorescence spectroscopy, extra virgin olive oil-EVOO, edible oils, adulteration, geographical origin, machine learning

## Abstract

In the present work, laser-induced breakdown spectroscopy (LIBS) and fluorescence spectroscopy are used and assessed for the detection of EVOOs’ adulteration with some non-EVOO edible oils (i.e., pomace, corn, sunflower, and soybean) and the discrimination of EVOOs based on geographical origin. For the direct comparison of the performance of the two techniques, the same set of EVOO samples was studied. The acquired spectroscopic data were analyzed by several machine learning algorithms, and the constructed predictive models are evaluated thoroughly for their reliability and robustness. In all cases, the high classification accuracies obtained support the potential and efficiency of both LIBS and fluorescence spectroscopy for the rapid, online, and in situ study of EVOOs’ authentication issues, with LIBS being more advantageous as it operates much faster.

## 1. Introduction

It is well known that extra virgin olive oil (EVOO) is an essential component of the Mediterranean diet, and it is highly recognized and preferred for its aroma, taste, and flavor, distinguishing it from other non-EVOO oils. Additionally, it is renowned for its health benefits, i.e., protection against chronic diseases, and its anti-inflammatory and antioxidant properties [1,2,3,4,5]. These characteristics are driving the high demand for olive oil [6], particularly extra virgin olive oil (EVOO), and encourage the increase of EVOO’s production. Nevertheless, the increasing demand for EVOO, is frequently followed by some malpractices, the most common ones being adulteration with different non-EVOO oils [7,8,9,10,11] and the mislabeling of geographical and/or varietal origins [12,13,14,15,16]. These malpractices hurt consumers’ confidence, who become more distrustful, thus preferring the purchase of certified olive oil products, as e.g., those characterized by protected designation of origin (PDO), protected geographical identification (PGI), etc., which have been established by the European Union (EU) (Regulation (EU) 2024/1143) [17]. Therefore, the development of alternative methods, capable of rapid and efficient operation to ensure olive oil authenticity is important.

In that view, various methods, including different well-established analytical chemistry techniques, are suggested for olive oil authentication [18,19,20]. For example, gas chromatography (GC) has been applied for the determination of fatty acids (FAs) and their trans-isomers, which are found in lower-quality oils, such as refined olive oil, that are often used as adulterants [21]. In addition, high-performance liquid chromatography (HPLC) has been employed for the determination of triglycerides (TAGs) in olive oil, which can serve as an indicator of olive oil adulteration [22,23]. However, TAGs’ analysis requires lengthy sample preparation procedures. Alternatively, HPLC-mass spectrometry has been employed for their measurements since it can detect them with higher sensitivity [24]. Besides olive oil’s adulteration detection, these techniques have also been proposed for the identification of olive oil’s geographical origin [25,26]. However, it should be noted that they all involve time-consuming sample preparation, costly equipment, and skilled personnel.

More recently, different spectroscopic techniques have also been proposed for similar tasks [27] and references therein, as for example laser-induced breakdown spectroscopy (LIBS) and fluorescence spectroscopy. LIBS technique, using a laser beam to induce microplasma on the surface of a sample and monitoring the plasma emission, is most often employed for the determination of the elemental composition of samples. LIBS can operate on samples of any physical state (i.e., liquids, gaseous, or solids) and provides rapid, online, and in situ measurements, without necessitating any prior sample preparation [28,29,30]. Fluorescence spectroscopy, using light from a conventional light source, typically a Xe arc lamp, can excite the different constituents of a sample, resulting in the emission of fluorescence, providing spectral fingerprint(s) that can serve for the identification and quantification of the chemical constituents of the sample [31,32,33]. In general, fluorescence spectroscopy does not require lengthy sample preparation [34,35,36]. However, some studies, as e.g., in the case of olive oil detection adulteration, have reported the need for sample preparation steps, such as dilution in n-hexane (1% *w*/*v*) [37,38] or spectrophotometrically pure 2,2,4-trimethylpentane [39]. Both techniques have been suggested and applied for diverse applications [40,41,42,43,44], including food authentication and quality control issues [45,46,47,48].

Caceres et al. [49] used LIBS and machine learning tools to investigate the possibility of the adulteration detection of some EVOOs blended with non-EVOO oils. They reported classification accuracies up to 95%. More recently, Nanou et al. employed LIBS [50], UV-Vis-NIR absorption spectroscopy [51], and machine learning to discriminate some EVOOs from their adulterated counterparts. They reported exceptionally high prediction accuracies, reaching up to 99%. Regarding the discrimination of EVOOs based on geographical origin, Gyftokostas et al. [52] employed LIBS assisted by machine learning, demonstrated the successful classification of the olive oil samples based on geographical origin, achieving excellent prediction accuracies and attaining values up to 100%. In another study by Gyftokostas et al. [53], LIBS was used to discriminate some EVOO samples from binary mixtures of them based on geographical origin. Again, excellent discrimination was obtained, with accuracy values yielding 100%.

As for the use of fluorescence spectroscopy concerning the detection of olive oil adulteration, Milanez et al. [36], having studied some EVOOs and their mixtures and employing different regression models, reported R^2^ values as high as 0.95–0.99. Similarly, Mu et al. [54], having investigated the discrimination of EVOOs from their mixtures with some non-EVOO oils, found excellent classification accuracies, i.e., up to 100%. In the same spirit, Jiménez-Carvelo et al. [55] reported that fluorescence spectroscopy was effective for the detection of adulteration of some Argentinean EVOOs, while their discrimination based on geographical origin was rather unsuccessful. In another work by Al Riza et al. [56], examining the classification of some Italian EVOOs based on geographical origin, an accuracy of ~90% was reported. In another study, Kontzedaki et al. [57] used fluorescence, absorption, and Raman spectroscopies for the classification of some Greek olive oils based on geographical origin and reported that fluorescence spectroscopy yielded a classification accuracy of ~82%.

In the above studies as well as in other literature works using LIBS and fluorescence spectroscopy, these techniques used different sets of olive oil samples. In fact, no study exists where both techniques study the same set of samples, to the best of our knowledge. In the current work, LIBS and fluorescence spectroscopy are employed for the detection of adulteration and the discrimination of the samples based on geographical origin, using the same samples for both techniques. In addition, for the analysis of the spectroscopic data, the same machine learning algorithms are employed and assessed. The present approach allows for direct comparison and comprehensive assessment of these techniques.

## 2. Materials and Methods

### 2.1. EVOOs and Non-EVOO Oil Samples

In the present work, 40 monovarietal EVOO samples of Koroneiki and Kolovi cultivars and mixtures of them with some non-EVOO edible oils are studied. The EVOOs were collected from local producers and cooperatives from four different geographical regions of Greece, including the islands of Crete and Lesvos, and the mainland, Messenia and Achaia. All samples were collected by mechanical harvesting and handpicking during the harvest season 2023–2024. For the adulteration of the pure EVOOs, 4 commercially available non-EVOO oils were used, i.e., pomace (PO), corn (CO), soybean (SBO), and sunflower (SFO) oils, purchased from the local market. One EVOO from each region, i.e., 4 EVOOs in total, was used and mixed with each of the non-EVOO oils, in different proportions ranging from 10 to 90% *w*/*w*, with a step of 10%. As a result, a total of 144 binary mixtures (i.e., adulterated EVOO samples) were prepared. After the mixtures were stirred for 10–15 min to ensure their homogeneity, they were stored in dark glass bottles and kept in a refrigerator at −2 to −4 °C. Prior to any measurement, they were left out overnight to reach ambient temperature. For the LIBS measurements, a small quantity of each sample (~1.5 mL) was placed in a shallow glass recipient, while for the fluorescence measurements, the sample was put in 1 cm pathlength glass cells.

### 2.2. Experimental Setups

The LIBS measurements were performed using the fundamental output at 1064 nm, of a 5 ns Q-switched Nd:YAG (Quanta-Ray INDI, Spectra Physics, Santa Clara, CA, USA), operating between 1–10 Hz. The laser beam had an energy of ~80 mJ, and it was focused on the sample’s surface via a planoconvex 150 mm quartz lens to induce a micro-plasma. The emitted radiation from the plasma was collected via a 50 mm fused-silica lens into a quartz fiber bundle, the latter being coupled to the entrance slit (10 μm) of an AvaSpec-ULS4096CL-EVO (Avantes, Apeldoorn, The Netherlands) spectrograph, equipped with 300 line/mm grating and a 4096 pixel CMOS detector. From these pixels, only 2751 were utilized, covering the 200–1000 nm spectral range. For the acquisition of the LIBS spectra, a time delay (t_d_) of 1.28 μs and an integration time (t_w_) of 1.05 ms were selected. For each sample, 10 consecutive laser shots were averaged, corresponding to one LIBS measurement, while 10 such LIBS measurements, at different locations on the surface of the sample, were collected. The collection time for these measurements lasted ~20 s.

For the fluorescence measurements, a FluoroMax-4 spectrofluorometer was used (Horiba Scientific, Jobin Yvon, Tokyo, Japan), equipped with a 150 W Xenon arc lamp. The measurement of the fluorescence (in the spectral region 362–800 nm) was performed by a photon-counting photomultiplier (R928P). The fluorescence spectra were acquired from 1 cm pathlength plastic cuvettes, employing right-angle geometry. The excitation wavelength was set at λex = 355 nm, while the bandpass of the two monochromators of the spectrofluorometer was adjusted to be 3 nm, both for the excitation and the emission. The scan speed and the integration time intervals were set at 2 nm and 0.1 s, respectively. For each sample, 10 measurements were taken, each measurement requiring 1 min, thus ~10 min in total.

### 2.3. Machine Learning Algorithms

For the classification tasks of the spectroscopic data, different machine learning algorithms were applied and evaluated, namely principal component analysis (PCA), linear discriminant analysis (LDA), support vector machines (SVMs), logistic regression (LR), and gradient boosting (GB) [58,59]. In brief, PCA, an unsupervised learning technique, allows for the reduction of the variables in a dataset by converting them into a smaller set of uncorrelated components, the so-called principal components (PCs), while maintaining most of the initial information (i.e., data variance). The rest algorithms employed, i.e., LDA, SVMs, LR, and GB, are supervised learning algorithms. LDA emphasizes class discrimination by identifying a projection direction that maximizes the ratio of inter-class variance to intra-class variance, making it particularly effective for classification tasks. SVMs can further enhance the classification by constructing a hyperplane that maximizes the margin between classes, using the support vectors, i.e., data points closest to the hyperplane, defining the decision boundaries. LR is a probabilistic classification approach, employing a logistic function to predict class probabilities while ensuring the outputs remain within the range of 0 to 1. Finally, GB, an ensemble learning method, builds iteratively a series of weak learners, such as decision trees, with each model correcting the errors of the previous one, finally leading to improved overall accuracy. All analyses were implemented using Python 3.8 [60], using essential libraries such as Scikit-Learn, Pandas, and NumPy supporting the modeling and the preprocessing steps. In addition, data standardization was performed by applying a standard scaler, which centered the features around a mean of zero and scaled them to unit variance.

For each one of the above algorithms, a corresponding predictive model was constructed, whose reliability was assessed by both internal and external validation. For this purpose, the datasets were split into training and testing subsets. For the internal validation, the k-fold cross-validation method was used, where the training dataset was divided into k subsets, and in each iteration, the k-1 subsets were used for training, while the remaining subset served for validation. This process was repeated k times (k = 10), providing an average accuracy score and a standard error. For the external validation, an independent subset of data, not previously used for the training, was used.

For the evaluation of the performance of each model, different metrics were calculated, such as the precision, the recall, and the overall accuracy (i.e., classification and prediction accuracy). Precision measures the accuracy of positive class predictions, while recall measures the ability to correctly identify all positive samples. The calculation of these metrics provides, in principle, a deeper insight into the strengths and limitations of each model in handling the data. Furthermore, for each model, the corresponding confusion matrix was constructed, where the correct and false predictions occurred were represented by the diagonal and the non-diagonal elements of the matrix, respectively.

### 2.4. Preparation of the Datasets

To perform the different classification tasks of interest in this work, the initial spectroscopic datasets were divided into training and test subsets, following an 80:20 ratio, to ensure the robust evaluation of the different models. The classification tasks examined included the discrimination of pure EVOOs from their binary mixtures with the non-EVOO oils, the identification of the type of non-EVOO oil used, and the classification of the pure EVOOs, the EVOO mixtures, and the pure EVOOs and their mixtures based on geographical origin.

Thus, for the discrimination of the pure EVOOs from their corresponding mixtures, the 40 pure EVOO samples were treated as one class, while the 144 EVOO/non-EVOO mixtures as a second class. The training set consisted of 144 samples (i.e., 32 EVOOs and 122 mixtures), and the test set comprised 40 samples (i.e., 8 EVOOs and 32 mixtures).

For the identification of the type of non-EVOO oil used for adulteration, two approaches were considered. At first, all 144 mixtures were distributed into four classes based on the non-EVOO oil used (pomace, corn, soybean, and sunflower oil), with each class comprising 36 samples. In this case, the training set contained 112 samples (i.e., 28 samples per adulterant), and the test set included 32 samples (i.e., 8 samples per non-EVOO adulterant). Next, the same task was performed in a more specific way, i.e., by examining the identification of the type of non-EVOO oil used separately for each geographical region. In this case, 36 samples were used for every region, with each class encompassing 9 adulterated EVOO samples. Therefore, the training set contained 28 samples (i.e., 7 samples per adulterant), and the test set included 8 samples (2 samples per adulterant).

For the classification of pure EVOOs based on geographical origin, the 40 EVOO samples were distributed in four classes (i.e., Crete, Lesvos, Messenia, and Achaia), each comprising 10 samples. From these, 32 samples (i.e., 8 from each region) were used to train the models, and 8 samples (i.e., 2 from each region) were used for test.

For the classification of the EVOO/non-EVOO mixtures based on geographical origin, the 144 mixtures (i.e., 36 samples for each geographical region), labeled as “Crete”, “Lesvos”, “Messenia”, and “Achaia”, were used. For this analysis, 112 samples (i.e., 28 from each region) were used for model training, and the remaining 32 samples (8 from each region) were used for test.

For the classification of both pure EVOOs and their mixtures based on geographical origin, all 184 samples were considered, forming four classes (corresponding to the four regions), with each class containing 46 samples (i.e., 10 EVOOs and 36 mixtures). The dataset was divided into 144 training samples (i.e., 8 EVOOs and 28 mixtures per class) and 40 test samples (i.e., 2 EVOOs and 8 mixtures per class).

Prior to the different analyses performed, the raw spectroscopic data from both LIBS and fluorescence spectroscopy were preprocessed using PCA for dimensionality reduction. The optimum number of PCs was selected to maximize the classification and prediction performances for each classification algorithm. The number of PCs used for each model is given in the corresponding tables in the Section 3.

## 3. Results

### 3.1. LIBS and Fluorescence Spectra Analysis

**Figure 1:**
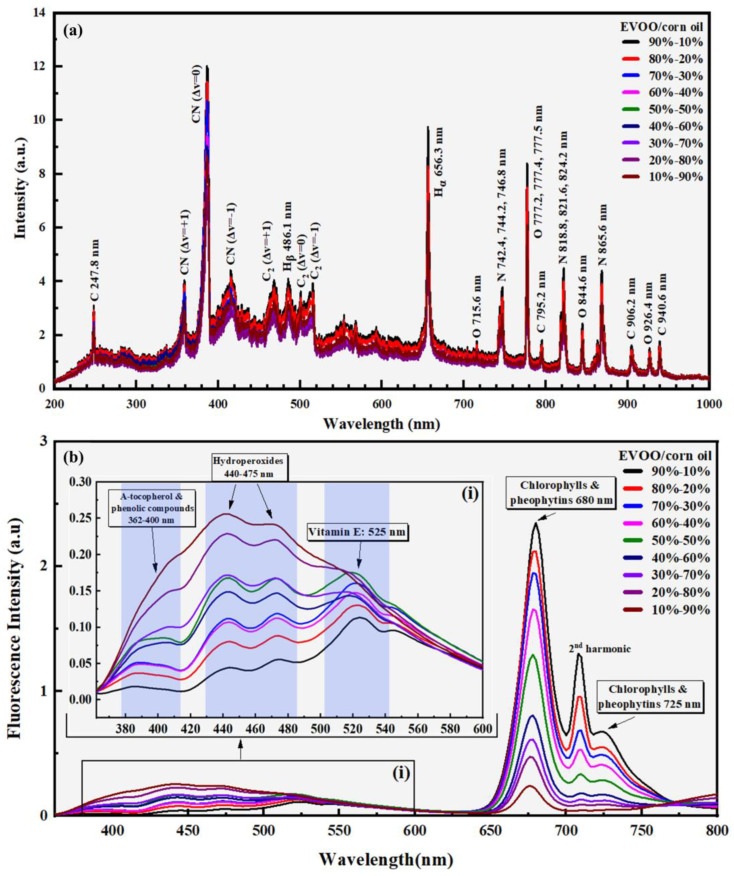
(**a**) LIBS and (**b**) fluorescence spectra of an EVOO sample (from the region of Achaia) mixed with commercial corn oil, in different proportions (from 10 to 90% *w*/*w*).

In Figure 1a, indicative LIBS spectra of an EVOO sample mixed with corn oil at different proportions are presented. As can be seen, the carbon C(I), oxygen O(I), nitrogen (N), and hydrogen (H_α_ and H_β_) atomic lines, and the vibrational band progressions of CN and C_2_ diatomic molecules were observed. The corresponding wavelengths of these emissions features are also shown in Figure 1a. Their assignments were based on the National Institute of Standards and Technology (NIST) spectral database [61] and previous works of our group [51,52].

The fluorescence spectra of the same EVOO mixtures are shown in Figure 1b. As can be seen in these spectra, most of the characteristic bands of olive oil constituents, usually observed in the fluorescence spectra of EVOOs, such as a-tocopherol and phenolic compounds (362–400 nm) [62], hydroperoxides (440–475 nm) [62,63], vitamin E (~525 nm) [63,64], chlorophylls (~680 nm) [63,64], and pheophytins (~725 nm) [64], were observed. The emission at ~710 nm, due to a leakage of the second harmonic of the excitation wavelength (~355 nm), was not related to the fluorescence of the EVOO and its mixtures, and therefore, it was excluded from the analysis of the spectra and the following discussion.

From a simple inspection of the fluorescence spectra, it was easily observed that as the corn content in the EVOO–corn mixtures increased, some of the observed bands were decreasing, while others exhibited the opposite trend. In fact, the intensity of the bands associated with the content of chlorophylls and pheophytins was monotonically decreasing, due to the decrease of the EVOO content in the binary mixtures, while the bands associated with the hydroperoxides, vitamin E, and phenolic compounds exhibited an increasing trend as expected for the non-EVOO edible oils (as can be seen in Supplementary Figure S1). On the contrary, in the LIBS spectra, an unclear trend in the intensities of the observed spectral features was observed. Nevertheless, this is well aligned with the fact that the LIBS emissions reflect the elemental composition of the samples, which remained relatively unchanged for the different mixtures, due to the very similar elemental content of the EVOOs and the non-EVOO oils, consisting basically of C, H, and O atoms. Although the chemical composition of the mixtures varies, increasing the corn oil content, the atomic elemental composition did not. The latter was also reflected in the LIBS spectra of EVOO samples from different geographical regions, as shown in Supplementary Figure S2a, where despite differences in climatic conditions, soil composition, etc., the elemental composition of the EVOOs remained unchanged. As a result, the LIBS spectra of these EVOOs exhibited only minor differences in the intensities of the spectral features. In contrast, the corresponding fluorescence spectra, reflecting the chemical composition of the samples, presented more obvious differences, particularly concerning the phenolic compounds, hydroperoxides, and vitamin E (as can be seen in Supplementary Figure S2b).

### 3.2. Discrimination of the Pure EVOOs from Their Mixtures with the Non-EVOO Oils

The discrimination of pure EVOOs from their mixtures with the non-EVOO oils was among the main goals of the present work. For this purpose, the 40 EVOOs were considered as one class, while the 144 EVOO/non-EVOO mixtures were considered another class. The classification and the prediction accuracies yielded by the different algorithms employed for this analysis are summarized in Table 1. As shown, both spectroscopic techniques resulted in excellent discrimination of the EVOO samples from their mixtures. In more detail, using the LIBS data, the LDA, SVMs, LR, and GB models attained classification and prediction accuracies as high as 100%, while the fluorescence data achieved classification and prediction accuracies between 95 and 99%. A more detailed picture of the classification results is provided by the corresponding confusion matrices presented in Supplementary Table S1. As can be seen, for the LIBS technique, all models predicted successfully all of the spectra of the EVOOs (80 spectra in total) and their mixtures (320 spectra in total), while the use of fluorescence data resulted in slightly lower classification rates; this small decrease was mainly observed for the pure EVOOs (from 2 to 20 spectra out of 80, depending on the model, were falsely classified). Moreover, the precision and recall scores attained values up to 1, both for LIBS, and fluorescence data (see Supplementary Table S1).

### 3.3. Identification of the Type of Non-EVOO Oil Used for EVOOs’Aadulteration

Following the successful discrimination of the EVOOs from their mixtures with non-EVOO oils, the possibility of identification of the type of non-EVOO oil used was investigated. This issue was examined from two different viewpoints. At first, considering all the 144 mixtures of the EVOOs from all geographical regions distributed to 4 classes based on the non-EVOO used as an adulterant and then separately considering the mixtures of EVOOs of each of the 4 geographical regions, each distributed to 4 classes based on the non-EVOO used. The latter investigation was performed to evaluate the effect of the different characteristics of the EVOOs originating from very different geographical regions, e.g., the islands and the mainland, as it is known that the soil, the climatic conditions, etc. can affect EVOOs’ characteristics.

**Figure 2:**
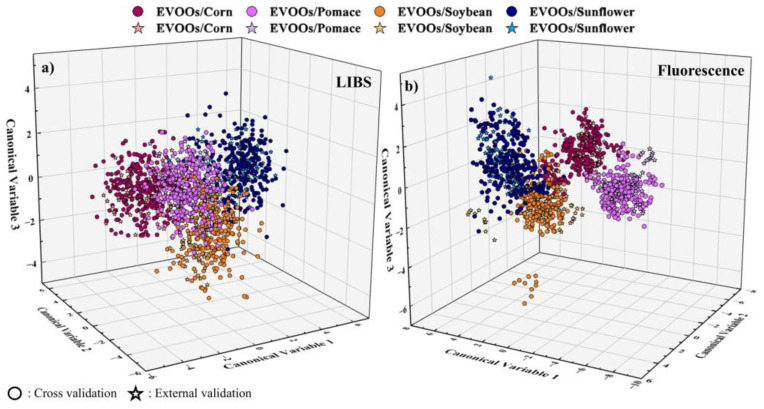
LDA plots showing the formation of classes based on the type of the non-EVOO oil, using: (**a**) the LIBS and (**b**) the fluorescence data.

For each approach, the classification and prediction accuracies were calculated for each model and are summarized in Table 2. As shown, the LIBS data resulted in classification and prediction accuracies up to ~86 and ~90%, respectively, while the fluorescence data resulted in relatively higher classification and prediction accuracies, ranging between 92 and 99% and between 90 and 99%, respectively, depending on the algorithmic model used. In addition, the confusion matrices for all models employed were prepared; they are presented in Supplementary Tables S2 and S3. As can be seen from these tables, in the case of LIBS data, the misclassifications were found to occur across all classes, particularly for the class of EVOOs mixed with soybean oil, where some spectra misclassified in the classes of EVOOs mixed with pomace oil, while using the fluorescence data, almost all spectra were successfully classified. In addition, the precision and recall scores were also determined, attaining values from 0.83 to 0.93 for LIBS and 0.76 to 1 for the fluorescence data (see Supplementary Tables S2 and S3). A visualization of these results, further facilitating their evaluation, is provided by the corresponding LDA plots, which are prepared and presented in Figure 2. As shown in these plots, the formation of the four classes was clearly observable, with some minor overlapping between them, for both the LIBS and the fluorescence data.

**Figure 3:**
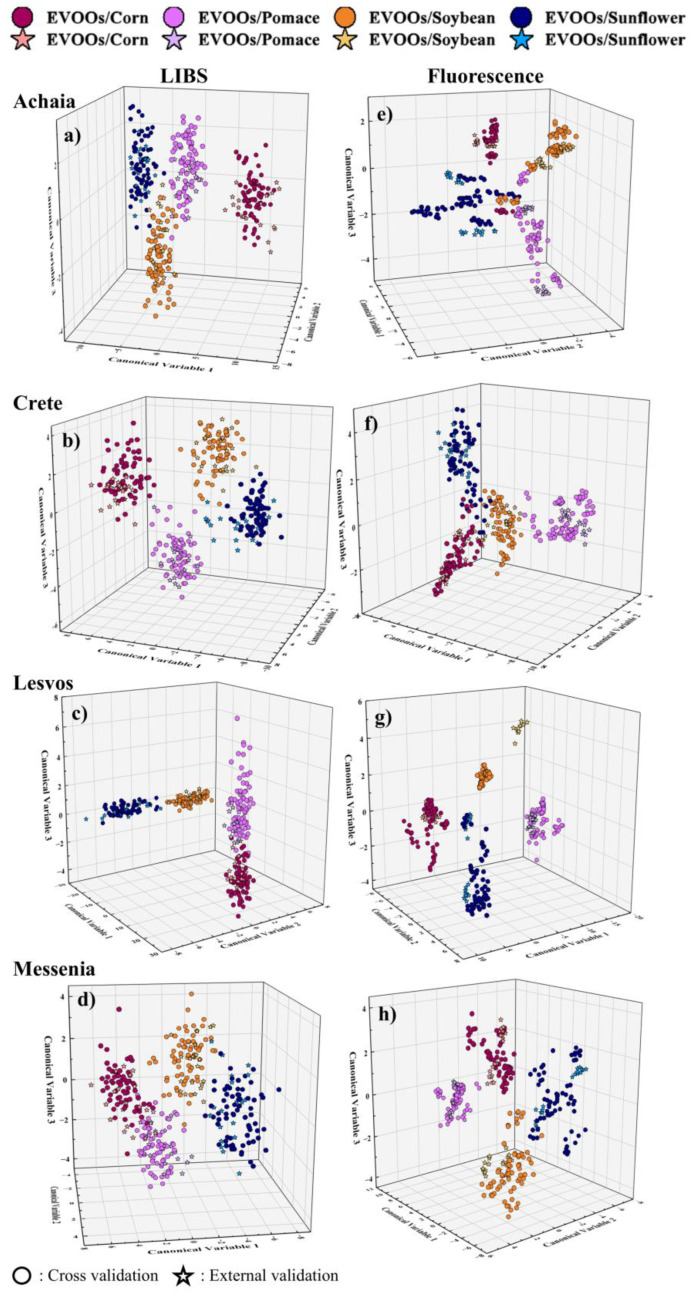
LDA plots showing the formation of the different classes based on the type of non-EVOO oil used, for the EVOOs of each geographical region using (**a**–**d**) the LIBS and (**e**–**h**) the fluorescence data.

Then, the case in which the EVOO mixtures of each geographical region were considered separately and distributed to four classes based on the non-EVOO used was examined. The results obtained for each region are presented in Table 3. Concerning the mixtures using EVOOs from the Achaia region, the LDA model yielded a classification accuracy of 99% for the LIBS data and 90% for the fluorescence data, while the prediction accuracies were up to 97% and 99%, respectively. Similarly, the SVM model applied to the LIBS and fluorescence data, achieved classification accuracies of 96% and 99% and prediction accuracies of 96% and 95%, respectively. The implementation of the LR algorithm resulted in classification accuracies of 95% and 98% and prediction accuracies of 96% and 92%, for LIBS and fluorescence data, respectively. The GB model reached an accuracy of 86% for the LIBS data and 99% for the data from the fluorescence measurements, while the prediction accuracies were found to be 89% and 90%, respectively. Similar quality results were obtained for the EVOOs of the other three geographical regions, as detailed in Table 3. The corresponding confusion matrices were also constructed and are presented in Supplementary Tables S4 and S5. As shown, most of the LIBS and fluorescence spectra were properly categorized, especially by the LDA, SVMs, and LR models, where in almost all cases, the spectra of pure EVOOs were correctly predicted (20 spectra in total), while the GB model exhibited a relatively larger number of misclassifications, as e.g., for the pure EVOOs, where 1–8 spectra, depending on the geographical origin, were misclassified. Regarding the precision and recall scores, the models yielded values ranging between 0.77 and 1 and between 0.60 and 1, respectively, for the LIBS data and 0.71–1 and 0.50–1, respectively, for the fluorescence data (see also Supplementary Tables S4 and S5). A better insight into the classification of these data is given by the LDA plots displayed in Figure 3. As shown, the respective classes are well formed in all cases, with those based on the fluorescence data exhibiting relatively more dispersion of the data points.

### 3.4. Classification of the EVOOs and Their Mixtures with No-EVOO Oils Based on the Geographical Origin of the EVOOs

The potential of LIBS and fluorescence spectroscopy in performing classification of EVOOs based on the geographical origin has been investigated for three cases: (i) for the pure EVOOs only, (ii) for the EVOOs’ mixtures only, and (iii) for both the EVOOs and their mixtures.

#### 3.4.1. Classification of EVOOs Based on Geographical Origin

**Figure 4:**
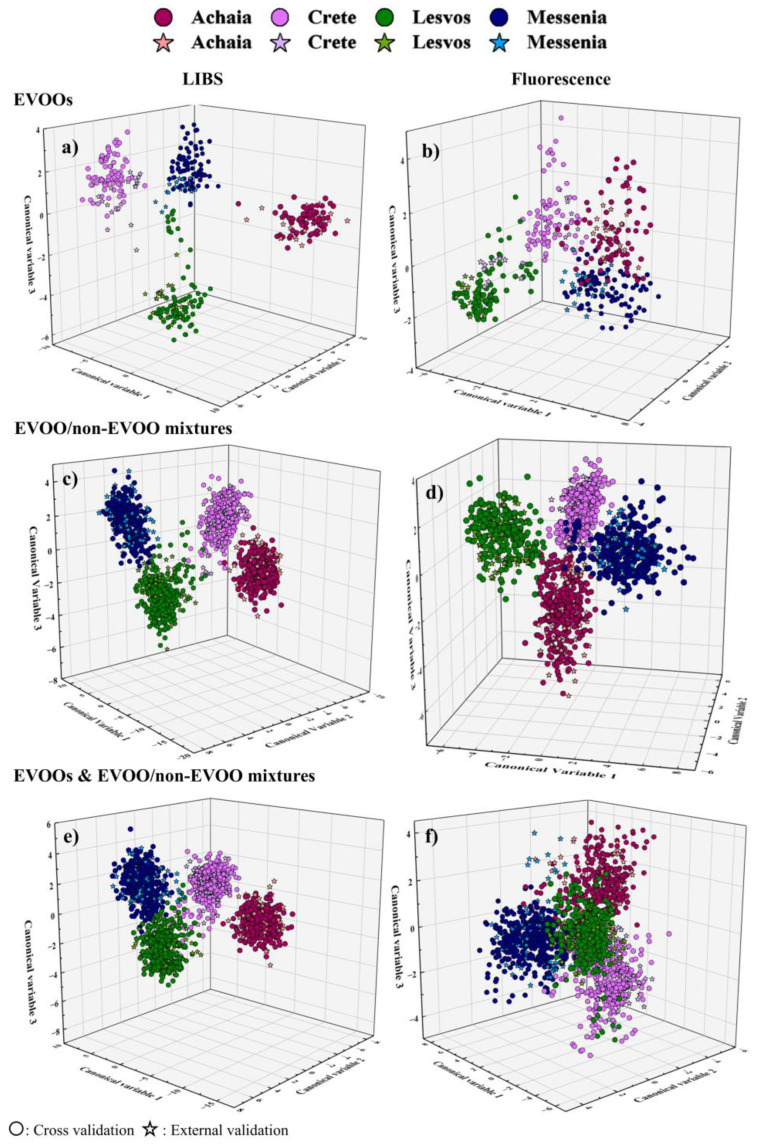
LDA plots showing the classification of the EVOOs and their mixtures based on geographical origin, using the LIBS and the fluorescence data, considering only the EVOOs (**a**,**b**), only the mixtures (**c**,**d**), and both the EVOOs and the mixtures (**e**,**f**).

In this case, the classification and prediction accuracies reached by all models were found to be better than 98% and 90%, respectively, with LDA, SVMs, and LR models yielding the best results. In the case of fluorescence data, the classification accuracies were very similar to those obtained using the LIBS data; however, the prediction accuracies were found to be significantly lower, with the LR algorithm reaching ~87%, the LDA and SVMs algorithms attaining ~80%, and the GB algorithm achieving only 61%. The corresponding confusion matrices are presented in Supplementary Table S6. As seen, the LIBS data were, in general, better classified compared to the fluorescence ones, with the latter resulting in higher rates of false predictions. In more detail, the LDA, SVMs, and LR models predicted correctly all LIBS spectra, with only one spectrum (out of 20) from Crete being incorrectly identified by SVMs, and LR, as belonging to Lesvos. As for the fluorescence spectra, some incorrect predictions occurred for all classes, particularly for Crete and Messenia. For example, 10 spectra out of 20 from Crete were categorized in the other classes. Additionally, the determined precision and recall scores were found to be 0.70–1 and 0.33–1 for LIBS and fluorescence data, respectively (see Supplementary Table S6). The LDA plots for each technique are displayed in Figure 4a,b. Notably, the LIBS results display smaller dispersion and more consistent class separation than those of the fluorescence measurements.

#### 3.4.2. Classification of EVOOs’ Mixtures Based on Geographical Origin

Next, the classification of the EVOOs’ mixtures based on the geographical origin was studied. The accuracies obtained from the analysis of the LIBS and fluorescence data were remarkably high, as can be seen in Table 4. In particular, the classification accuracies were up to ~98% for the LIBS data and up to ~99% for the fluorescence ones, while the prediction accuracies attained values up to ~99%, in both cases. The corresponding confusion matrices, providing more detailed information on the correct/false predictions, are presented in Supplementary Table S7. As it can be observed in these matrices most of the spectra were correctly predicted. Specifically, the LDA, SVMs, and LR models made one or two incorrect predictions both for the LIBS and fluorescence data, with the GB model exhibiting a slightly higher number of misclassifications in the case of LIBS, i.e., up to 5 spectra (out of 80 spectra in total), which can be considered negligible to its overall performance. The findings are also supported by the precision and recall scores, presented in Supplementary Table S7, where it is shown that values up to 1 for both spectroscopic datasets were obtained. Furthermore, the corresponding LDA plots, illustrated in Figure 4c,d, fully confirmed the successful discrimination of the mixtures in four classes, according to the geographical origin of the EVOO.

#### 3.4.3. Classification of the EVOOs and Their Mixtures with Non-EVOO Oils Based on Geographical Origin

Finally, the classification of both pure EVOOs and their mixtures based on geographical origin was examined. The classification and prediction accuracies were calculated, and they are summarized in Table 4. In more detail, for the LIBS data, the classification accuracies attained values up to ~98%, and the prediction ones reached exceptionally high values, i.e., up to 100%, in some cases. The corresponding accuracies for the fluorescence data, although they were found to be slightly lower, in general, they are still quite satisfactory. Specifically, the classification accuracies ranged between 93 and 99%, and the prediction accuracies were between 86 and 98% (see Table 4). From the confusion matrices constructed and presented in Supplementary Table S8, it becomes obvious that the vast majority of the spectra were correctly categorized. The precision and recall scores were also calculated and are provided in Supplementary Table S8, where it can be seen that values up to 1 were obtained. As seen, for the LIBS data, values of 0.77–1 were obtained, while for the fluorescence data, the values were 0.82–1. Lastly, the LDA plots depicted in Figure 4e,f, show that the four classes are more clearly separated in the LIBS data than in the case of fluorescence data.

## 4. Discussion

In the present study, LIBS and fluorescence spectroscopy techniques were employed, and their results were compared in detail for the detection of adulteration of EVOOs from four different geographical regions, with four non-EVOO edible oils, as e.g., the pomace, corn, soybean, and sunflower oils, often being used for EVOOs’ adulteration. The four geographical regions were selected based on their significance regarding quality, brand reputation, and volume of olive oil production. Furthermore, the LIBS and fluorescence spectroscopy data were analyzed to explore different classification/discrimination scenarios based on geographical origin. In that view, the discrimination of (i) the pure EVOOs, (ii) their mixtures with the non-EVOO oils, and (iii) both EVOOs and their mixtures based on geographical origin were explored separately. It is important to note that although both spectroscopic techniques have been employed in the past for the same or similar purposes, this is the first study, to our knowledge, where they are applied to the same set of EVOO and non-EVOO samples.

Initially, the discrimination between the pure EVOOs and the adulterated EVOOs was studied. The results obtained from the analysis of the LIBS and fluorescence data were remarkably successful, attaining prediction accuracies up to 100 and 99%, respectively. Next, the determination of the type of non-EVOO edible oil used as an adulterant was studied, following two approaches: considering the mixtures with each non-EVOO oil from all geographical regions as one class and then considering the adulterated EVOO samples of each geographical region distributed in four separate classes. Regarding the first approach, the different models using the LIBS data resulted in prediction accuracies of up to 90%, while using the fluorescence data, prediction accuracies of up to 98% were achieved. As far as the results of the second approach, both LIBS and fluorescence data resulted in prediction accuracies as high as 100%.

Regarding the capability of LIBS and fluorescence spectroscopy techniques for the discrimination of the EVOOs and their mixtures based on the geographical origin of the EVOOs, three different scenarios were examined: the classification of the EVOOs only, the EVOO/non-EVOO mixtures only, and the EVOOs and their mixtures together. In the first case, the LIBS technique achieved excellent results, with prediction accuracies reaching 100% for some of the models, while fluorescence spectroscopy was less efficient, attaining prediction accuracies between 61 and 80%. Concerning the second approach, both techniques succeeded in excellent prediction accuracies, up to 100%. Last, when analyzing the combined data from the EVOOs and their mixtures with the non-EVOO oils, both techniques yielded very successful results, with the prediction accuracies attaining 95–100% for the LIBS data and 86–98.5% for the fluorescence data. Overall, while both LIBS and fluorescence spectroscopy demonstrated excellent predictive capabilities, a notable difference between the two techniques lies in the time required by each technique. LIBS provided rapid measurements, requiring only a few seconds per sample, whereas fluorescence spectroscopy required several minutes.

It is useful at this point, to compare the present results with results from other studies appeared in the literature using LIBS or luorescence spectroscopy to study similar issues of olive oil authentication. Concerning the detection of EVOOs’ adulteration, Meng et al. [65] studied mixtures of EVOOs with soybean oil (in mixing ratios 5–50 *w*/*w*), employing three different spectroscopies, namely Fourier-transform infrared (FTIR), UV-Vis-NIR absorption, and fluorescence spectroscopies assisted by partial least squares discriminant analysis (PLS-DA). Accuracies up to 100% were reported using the FTIR and UV-Vis-NIR data and 73% for the fluorescence data. In the same spirit, Wang et al. [66] also studied mixtures of EVOOs with soybean oil with fluorescence and Raman spectroscopies. The analysis of the fluorescence and Raman data was performed using a multiple linear regression (MLR) model and resulted in R^2^ values of 0.80 and 0.99, respectively. Similarly, Milanez et al. [36] studied the detection of EVOO adulteration with soybean oil (in mixing ratios 10–300 g/kg) via fluorescence and UV-Vis-NIR absorption spectroscopies. For the analysis, multiple regression models were used; most of them yielded R^2^ values of 0.99.

Zhang et al. [67] used laser-induced fluorescence spectroscopy to study the detection of the adulteration of EVOOs with peanut and soybean oils (in mixing ratios 10 to 90%), employing MLR and partial least square regression (PLSR). R^2^ values of 0.99 were reported for both models. In a similar direction, Mu et al. [54] used fluorescence spectroscopy to study mixtures of EVOOs with peanut and rapeseed oils (in mixing ratios 2.5 to 50%). Having employed SVMs, artificial neural networks (ANN), and PLSR, classification accuracies of 100% were reported for the first two models, and an R^2^ of 0.99 was recorded for PLSR. In another study by Ali et al. [35], the detection of adulteration of some EVOOs with sunflower oil (in mixing ratios 5 to 95%) was conducted by fluorescence spectroscopy. The PLSR model used reached an R^2^ of 0.99.

Regarding the LIBS-based studies of adulteration detection, Caceres et al. [49] studied mixtures of EVOOs with sunflower, hazelnut, and corn oils (in mixing ratios 1–5% *v*/*v*). Using neural networks (NN), accuracies up to 95% were attained. In the same direction, Nanou et al. [50,51] studied the detection of adulteration of EVOOs with pomace, corn, soybean, and sunflower oils (in mixing ratios 10 to 90% *w*/*w*), employing LIBS and UV-Vis-NIR absorption spectroscopy; for the analysis, the LDA, SVMs, LR, and GB algorithms were implemented. In both studies, prediction accuracies ranging between 92% and 99% were reported.

As for the EVOOs’ classification based on geographical origin, Al Riza et al. [56] studied some Italian EVOOs by fluorescence spectroscopy; the spectroscopic data were analyzed by different machine learning models (i.e., LDA, SVMs, Complex Tree, and Fine k-Nearest Neighbor (k-NN)). It was reported that all algorithmic models employed attained accuracies lower than 70%, except for the k-NN, which yielded accuracies of ~90%. Along the same lines, Jiménez-Carvelo et al. [55] used near-infrared (NIR) and fluorescence spectroscopy to discriminate some Argentinean EVOOs based on geographical origin. For the analysis of the data, partial least square discriminant analysis (PLS-DA) was employed. It was reported that NIR spectroscopy could effectively classify the EVOO samples, while fluorescence spectroscopy was less satisfactory. In another study, Kontzedaki et al. [57] used fluorescence, UV-Vis-NIR absorption, and Raman spectroscopies for the discrimination of some Greek EVOOs based on geographical origin. The PLS-DA classification of the UV-Vis-NIR absorption and Raman data resulted in ~97 and ~94% accuracies, respectively, while fluorescence spectroscopy did not exceed ~82%.

Concerning the use of LIBS for the same classification task, Gazeli et al. [68] applied LIBS with LDA, SVMs, and Random Forest (RF) algorithms to classify EVOOs based on geographical origin and acidity, achieving accuracies between 90% and 99%. Similarly, Gyftokostas et al. [52,53] investigated the identification of the geographical origin of some Greek EVOOs using LIBS and applying different algorithmic models (i.e., LDA, Extremely Randomized Trees Classifier (ERTC), RF, eXtreme Gradient Boosting Classifier (XGBoost), SVMs). In both studies, high prediction accuracies up to 100% were obtained.

From the critical evaluation of the above presented results, it appears that fluorescence spectroscopy is more successful for EVOOs’ adulteration detection than for the discrimination of EVOOs based on geographical origin, while LIBS exhibits equally successful performance for both tasks. In addition, considering the much shorter time required to perform the LIBS measurements compared to that required to perform the fluorescence measurements, it becomes obvious that the LIBS technique is clearly more advantageous for rapid, in situ, and/or online applications.

## 5. Conclusions

In this work, a comparative study of EVOOs’ authentication was performed by employing LIBS and Fluorescence spectroscopy techniques and studying the same EVOO samples. For the analysis of the spectroscopic data different machine learning algorithms were used and assessed.

Both LIBS and fluorescence spectroscopy techniques were highly efficient to discriminate pure EVOOs from their mixtures with other non-EVOO edible oils, with LIBS being slightly more efficient. Fluorescence spectroscopy, on the other hand, was found to perform better for the identification of the type of the non-EVOO edible oil used for adulteration of the EVOOs, yielding prediction accuracies higher by ~10%, than LIBS. Concerning the geographical origin-based classification, LIBS was found to be more efficient, particularly in the case of pure EVOOs. The above-described systematic comparison and evaluation of both techniques across diverse tasks highlights both techniques’ efficiency, offering a robust framework for EVOOs’ authentication. However, LIBS seems to be better suited for rapid, in situ, and/or online quality control and authentication tasks as it can operate much faster.

## Figures and Tables

**Table 1:** Classification and prediction accuracies obtained by the different algorithms, using the LIBS and fluorescence data, for the discrimination of pure EVOOs from their mixtures, where all EVOOs were treated as one class, and all mixtures as another one.

Algorithm	LIBS			Fluorescence Spectroscopy		
	Classification (%)	No. of PCs	Prediction (%)	Classification (%)	No. of PCs	Prediction (%)
LDA	99.9 ± 0.2	60	100	93.2 ± 2.5	30	98.8
SVMs	100.0 ± 0.0	40	100	97.2 ± 1.6	10	97.5
LR	99.9 ± 0.2	30	100.0	99.0 ± 0.6	60	98.0
GB	99.5 ± 0.6	50	99.8	99.5 ± 0.6	20	95

**Table 2:** Classification and prediction accuracies obtained by the different algorithms, using the LIBS and the fluorescence data, for the identification of the type of the non-EVOO oil used, considering one class for each adulterant (i.e., four classes in total).

Algorithm	LIBS			Fluorescence Spectroscopy		
	Classification (%)	No. of PCs	Prediction (%)	Classification (%)	No. of PCs	Prediction (%)
LDA	85.2 ± 2.3	140	88.7	92.2 ± 1.2	30	98.1
SVMs	84.6 ± 3.0	80	90.0	95.5 ± 1.2	30	98.1
LR	83.1 ± 3.1	120	89.4	96.5 ± 2.2	50	99.4
GB	85.6 ± 3.1	90	87.2	99.6 ± 0.3	20	90.9

**Table 3:** Classification and prediction accuracies yielded by the different algorithms, using the LIBS and the fluorescence data for the EVOOs of each geographical region for the identification of the type of the non-EVOO oil used, considering one class for each adulterant (i.e., four classes in total).

Algorithm	LIBS			Fluorescence Spectroscopy		
	Classification (%)	No. of PCs	Prediction (%)	Classification (%)	No. of PCs	Prediction (%)
**Achaia**						
LDA	98.9 ± 2.3	30	97.5	90.0 ± 6.7	10	98.7
SVMs	95.7 ± 3.5	60	96.2	98.9 ± 1.6	10	95.0
LR	95.4 ± 3.6	110	96.3	97.9 ± 2.4	10	92.5
GB	85.7 ± 5.5	20	88.7	99.1 ± 0.6	10	90.0
**Crete**						
LDA	100.0 ± 0.0	40	100.0	98.9 ± 1.6	10	100.0
SVMs	99.3 ± 1.4	90	100.0	100.0 ± 0.0	10	100.0
LR	98.6 ± 1.7	50	100.0	100.0 ± 0.0	10	100.0
GB	89.6 ± 5.9	30	90.0	100.0 ± 0.0	10	85.0
**Lesvos**						
LDA	96.1 ± 4.6	30	95.0	100.0 ± 0.0	10	98.7
SVMs	95.0 ± 3.6	30	93.7	99.6 ± 1.1	10	100.0
LR	94.3 ± 3.6	20	97.5	100.0 ± 0.0	10	100.0
GB	94.6 ± 4.0	30	87.5	99.3 ± 1.5	10	87.5
**Messenia**						
LDA	98.8 ± 3.1	40	96.2	99.6 ± 1.1	10	100.0
SVMs	95.4 ± 3.2	40	96.2	100.0 ± 0.0	10	100.0
LR	94.3 ± 2.9	80	97.5	100.0 ± 0.0	10	100.0
GB	85.4 ± 6.1	30	92.5	98.6 ± 0.6	10	87.3

**Table 4:** Classification and prediction accuracies of the classification of the pure EVOOs, mixtures, and both EVOOs and their mixtures, based on geographical origin obtained by the different algorithms using the LIBS and the fluorescence data.

Approach	Algorithm	LIBS			Fluorescence Spectroscopy		
		Classification (%)	No. of PCs	Prediction (%)	Classification (%)	No. of PCs	Prediction (%)
**Only pure EVOOs**	LDA	98.8 ± 2.9	20	100.0	97.5 ± 1.9	30	80.0
	SVMs	100.0 ± 0.0	30	98.8	99.4 ± 1.2	40	80.0
	LR	100.0 ± 0.0	40	98.7	98.4 ± 2.1	60	87.5
	GB	98.1 ± 3.2	30	90.0	98.1 ± 2.5	30	61.3
**Only mixtures**	LDA	97.8 ± 1.8	170	99.4	97.5 ± 1.6	80	99.7
	SVMs	98.1 ± 0.9	190	99.4	99.0 ± 0.8	20	99.7
	LR	97.7 ± 1.0	170	99.1	98.9 ± 1.0	30	100.0
	GB	94.9 ± 2.2	190	94.4	99.7 ± 0.4	50	99.4
**EVOOs and mixtures**	LDA	98.8 ± 0.9	110	99.0	93.5 ± 2.2	70	95.0
	SVMs	98.4 ± 1.3	110	100.0	97.8 ± 0.8	30	98.5
	LR	97.3 ± 1.3	110	98.2	95.9 ± 1.5	70	97.2
	GB	96.6 ± 1.9	60	95.3	99.6 ± 0.6	50	86.3

## Data Availability

The original contributions presented in this study are included in the article/Supplementary Materials. Further inquiries can be directed to the corresponding author.

## Supplementary Materials

The following supporting information can be downloaded at: https://www.mdpi.com/article/10.3390/foods14061045/s1, Figure S1. Fluorescence spectra of EVOO samples, corn, pomace, soybean, and sunflower oils; Figure S2. (a) LIBS and (b) fluorescence spectra of EVOO samples from Achaia, Crete, Lesvos, and Messenia; Table S1. Confusion matrices and precision and recall scores for the different algorithms, using the LIBS and the fluorescence data, where all EVOOs were treated as one class, and all mixtures as another one; Table S2. Confusion matrices and precision and recall scores for the different algorithms, using the LIBS data, for the identification of the type of adulterant, considering one class for each adulterant (i.e., four classes in total); Table S3. Confusion matrices and precision and recall scores for the different algorithms, using the fluorescence data, for the identification of the type of adulterant, considering one class for each adulterant (i.e., four classes in total); Table S4. Confusion matrices and precision and recall scores for the different algorithms, using the LIBS data, for each geographical region, considering one class for each adulterant (i.e., four classes in total); Table S5. Confusion matrices and precision and recall scores for the different algorithms, using the fluorescence data, for each geographical region, considering one class for each adulterant (i.e., four classes in total); Table S6. Confusion matrices and precision and recall scores for the different algorithms, using the LIBS, and the fluorescence data, for the geographical origin discrimination of pure EVOOs; Table S7. Confusion matrices and precision and recall scores for the different algorithms, using the LIBS, and the fluorescence data, for the geographical origin discrimination of EVOO/non-EVOO mixtures; Table S8. Confusion matrices and precision and recall scores for the different algorithms, using the LIBS, and the fluorescence data, for the geographical origin discrimination of EVOOs and their mixtures.
