# Supplementary material for: Laser-Induced Breakdown Spectroscopy vs. Fluorescence Spectroscopy for Olive Oil Authentication
Source: Foods. 2025 Mar 19;14(6):1045. doi: 10.3390/foods14061045 (PMC11942084; doi:10.3390/foods14061045)
Supplement: Supplementary file 1 [file foods-14-01045-s001.zip › foods-3498948-supplementary.pdf]

Supplementary Material

Laser-Induced Breakdown Spectroscopy vs. Fluorescence Spectroscopy for Olive Oil Authentication

Marios Bekogianni [1], Theodoros Stamatoukos [1], Eleni Nanou [1,2] and Stelios Couris [1,2,*]

1 Department of Physics, University of Patras, 26504 Patras, Greece; up1068704@ac.upatras.gr (M.B.); up1071016@ac.upatras.gr (T.S.); e.nanou@iceht.forth.gr (E.N.)
2 Institute of Chemical Engineering Sciences (ICE-HT), Foundation for Research and Technology-Hellas (FORTH), 26504 Patras, Greece
* Correspondence: couris@upatras.gr; Tel.: +30-2610996086

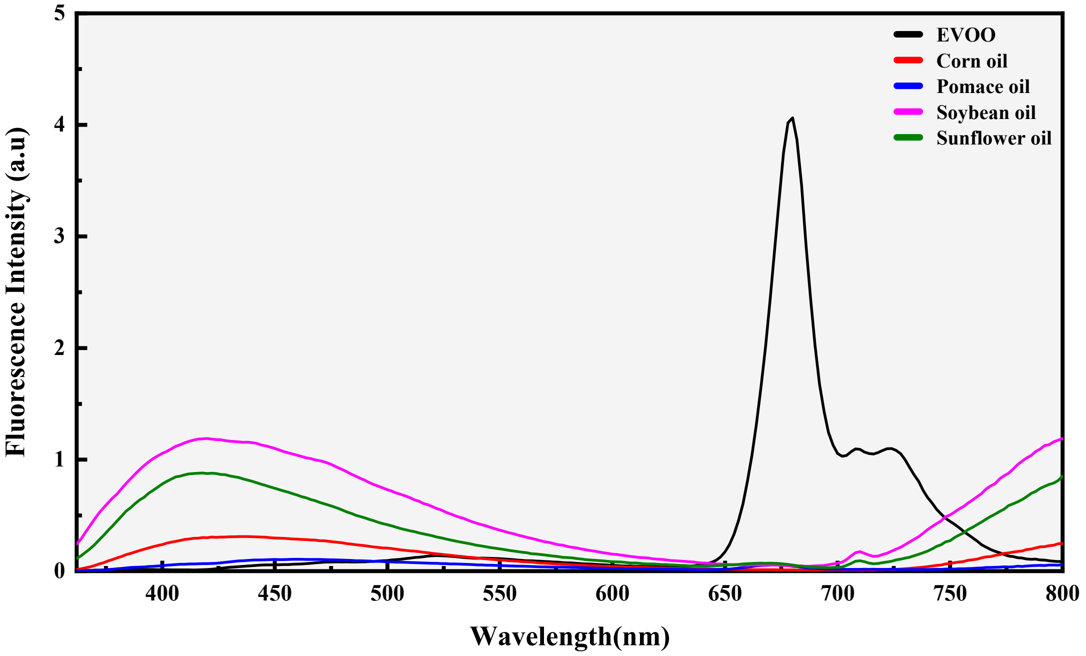

Figure S1. Fluorescence spectra of EVOO samples, corn, pomace, soybean, and sunflower oils.

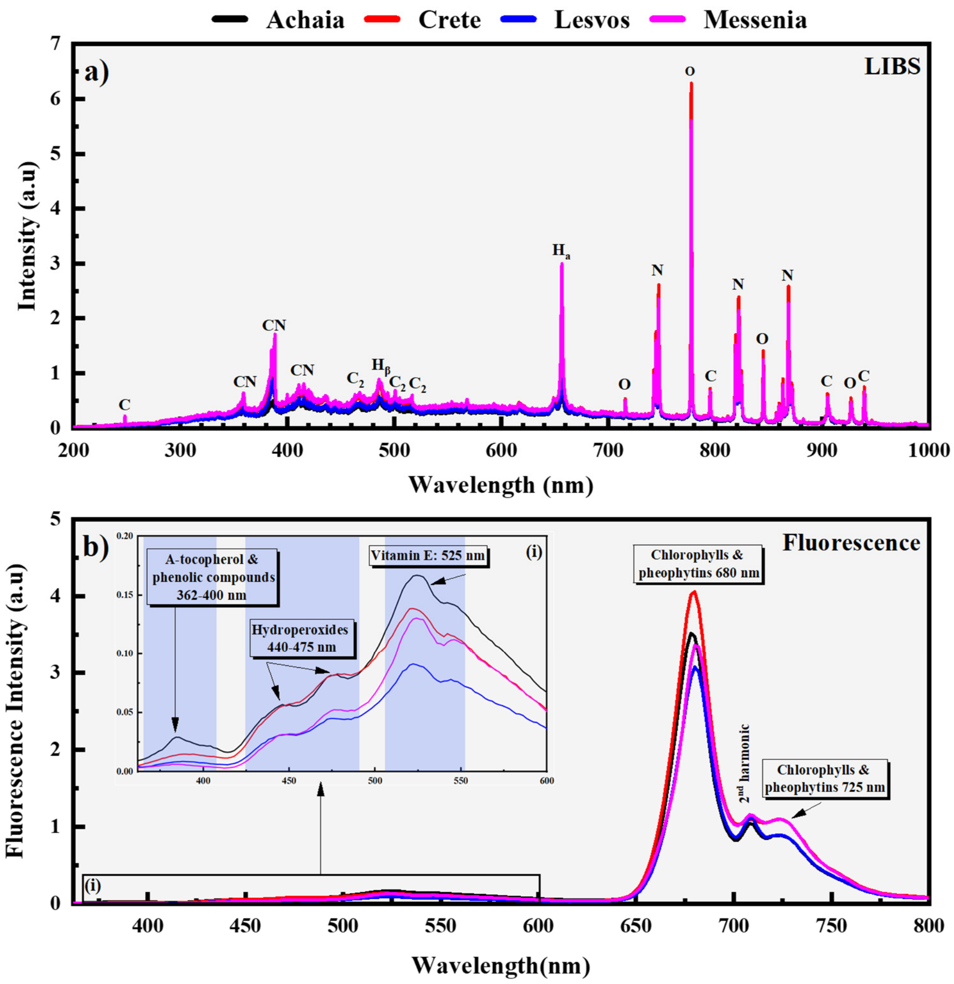

Figure S2. a) LIBS and b) fluorescence spectra of EVOO samples from Achaia, Crete, Lesvos, and Messenia.

Table S1. Confusion matrices and precision and recall scores for the different algorithms, using the LIBS and the fluorescence data, where all EVOOs were treated as one class, and all mixtures as another one.

**LIBS**								
	**LDA algorithm**				**SVMs algorithm**			
	**Predicted classes**				**Predicted classes**			
**Actual classes**	EVOOs	Mixtures	**Precision**	**Recall (Sensitivity)**	EVOOs	Mixtures	**Precision**	**Recall (Sensitivity)**
EVOOs	80	0	1	0.99	80	0	1	0.99
Mixtures	0	320	1	1	0	320	1	1
	**LR algorithm**				**GB algorithm**			
EVOOs	80	0	1	1	79	1	1	0.99
Mixtures	0	320	1	1	0	320	1	1
**Fluorescence spectroscopy**								
	**LDA algorithm**				**SVMs algorithm**			
EVOOs	78	2	1	0.88	70	10	1	0.99
Mixtures	3	317	0.97	1	0	320	1	1
	**LR algorithm**				**GB algorithm**			

EVOOs	72	8	0.94	1	60	20	1	0.99
Mixtures	0	320	1	0.98	0	320	1	1

Table S2. Confusion matrices and precision and recall scores for the different algorithms, using the LIBS data, for the identification of the type of adulterant, considering one class for each adulterant (i.e., four classes in total).

**LIBS**						
	**Predicted classes**					
**LDA algorithm**						
**Actual classes**	EVOO/CO mixtures	EVOO/PO mixtures	EVOO/SBO mixtures	EVOO/SFO mixtures	**Precision**	**Recall (Sensitivity)**
EVOO/CO mixtures	**71**	6	2	1	0.88	0.89
EVOO/PO mixtures	5	**71**	4	0	0.86	0.89
EVOO/SBO mixtures	4	6	**66**	4	0.88	0.82
EVOO/SFO mixtures	1	0	3	**76**	0.94	0.95
**SVMs algorithm**						
EVOO/CO mixtures	**74**	3	2	1	0.91	0.93
EVOO/PO mixtures	4	**74**	2	0	0.86	0.93
EVOO/SBO mixtures	2	9	**68**	1	0.86	0.85
EVOO/SFO mixtures	1	0	7	**72**	0.97	0.90
**LR algorithm**						
EVOO/CO mixtures	**69**	5	4	2	0.91	0.86
EVOO/PO mixtures	3	**73**	3	1	0.85	0.91
EVOO/SBO mixtures	3	8	**68**	1	0.87	0.85
EVOO/SFO mixtures	1	0	3	**76**	0.95	0.95
**GB algorithm**						
EVOO/CO mixtures	**64**	6	9	1	0.91	0.80
EVOO/PO mixtures	2	**72**	3	3	0.85	0.90
EVOO/SBO mixtures	0	6	**71**	3	0.83	0.89

EVOO/SFO mixtures	4	1	3	**72**	0.91	0.90

Table S3. Confusion matrices and precision and recall scores for the different algorithms, using the fluorescence data, for the identification of the type of adulterant, considering one class for each adulterant (i.e., four classes in total).

**Fluorescence spectroscopy**						
**Predicted classes**						
**LDA algorithm**						
**Actual classes**	EVOO/CO mixtures	EVOO/PO mixtures	EVOO/SBO mixtures	EVOO/SFO mixtures	**Precision**	**Recall (Sensitivity)**
EVOO/CO mixtures	**80**	0	0	0	1	1
EVOO/PO mixtures	0	**80**	0	0	1	1
EVOO/SBO mixtures	0	0	**74**	6	1	0.93
EVOO/SFO mixtures	0	0	0	**80**	0.93	1
**SVMs algorithm**						
EVOO/CO mixtures	**80**	0	0	0	0.99	1
EVOO/PO mixtures	0	**80**	0	0	1	1
EVOO/SBO mixtures	0	0	**75**	**5**	1	0.94
EVOO/SFO mixtures	1	0	0	**79**	0.94	0.99
**LR algorithm**						
EVOO/CO mixtures	**80**	0	0	0	1	1
EVOO/PO mixtures	0	**80**	0	0	1	1
EVOO/SBO mixtures	0	0	**78**	2	1	0.97
EVOO/SFO mixtures	0	0	0	**80**	0.98	1
**GB algorithm**						
EVOO/CO mixtures	**80**	0	0	0	1	1
EVOO/PO mixtures	0	**80**	0	0	1	1
EVOO/SBO mixtures	0	0	**70**	10	0.79	0.88
EVOO/SFO mixtures	0	0	19	**61**	0.86	0.76

Table S4. Confusion matrices and precision and recall scores for the different algorithms, using the LIBS data, for each geographical region, considering one class for each adulterant (i.e., four classes in total).

**LIBS**												
**Achaia**												
	**LDA algorithm**						**SVMs algorithm**					
	**Predicted classes**						**Predicted classes**					
**Actual classes**	EVOO/CO	EVOO/PO	EVOO/SBO	EVOO/SFO	**Precision**	**Recall (Sensitivity)**	EVOO/CO	EVOO/PO	EVOO/SBO	EVOO/SFO	**Precision**	**Recall (Sensitivity)**
EVOO/CO	**20**	0	0	0	1	1	**20**	0	0	0	1	1
EVOO/PO	0	**19**	1	0	1	0.95	0	**19**	1	0	0.95	0.95
EVOO/SBO	0	0	**19**	1	0.95	0.95	0	1	**18**	1	0.95	0.90
EVOO/SFO	0	0	0	**20**	0.95	1	0	0	0	**20**	0.95	1
	**LR algorithm**						**GB algorithm**					
	EVOO/CO	EVOO/PO	EVOO/SBO	EVOO/SFO			EVOO/CO	EVOO/PO	EVOO/SBO	EVOO/SFO		
EVOO/CO	**20**	0	0	0	0.95	1	**20**	0	0	0	1	1
EVOO/PO	0	**20**	0	0	0.89	0.85	0	**16**	4	0	0.80	0.80
EVOO/SBO	0	2	**17**	1	0.87	1	0	4	**16**	0	0.76	0.80
EVOO/SFO	0	0	0	**20**	1	0.85	0	0	1	**19**	1	0.95
**Crete**												
	**LDA algorithm**						**SVMs algorithm**					
	EVOO/CO	EVOO/PO	EVOO/SBO	EVOO/SFO			EVOO/CO	EVOO/PO	EVOO/SBO	EVOO/SFO		
EVOO/CO	**20**	0	0	0	1	1	**20**	0	0	0	1	1
EVOO/PO	0	**20**	0	0	1	1	0	**20**	0	0	1	1
EVOO/SBO	0	0	**20**	0	1	1	0	0	**20**	0	1	1
EVOO/SFO	0	0	0	**20**	1	1	0	0	0	**20**	1	1
	**LR algorithm**						**GB algorithm**					
	EVOO/CO	EVOO/PO	EVOO/SBO	EVOO/SFO			EVOO/CO	EVOO/PO	EVOO/SBO	EVOO/SFO		
EVOO/CO	**20**	0	0	0	1	1	**19**	1	0	0	0.95	0.95
EVOO/PO	0	**20**	0	0	1	1	1	**17**	1	1	0.77	0.85
EVOO/SBO	0	0	**20**	0	1	1	0	3	**17**	0	0.94	0.85
EVOO/SFO	0	0	0	**20**	1	1	0	1	0	**19**	0.95	0.95
**Lesvos**												
	**LDA algorithm**						**SVMs algorithm**					
	EVOO/CO	EVOO/PO	EVOO/SBO	EVOO/SFO			EVOO/CO	EVOO/PO	EVOO/SBO	EVOO/SFO		
EVOO/CO	**18**	2	0	0	0.95	0.90	**17**	3	0	0	0.94	0.85
EVOO/PO	1	**19**	0	0	0.90	0.95	1	**19**	0	0	0.86	0.95
EVOO/SBO	0	0	**20**	0	0.95	1	0	0	**20**	0	0.95	1
EVOO/SFO	0	0	1	**19**	1	0.95	0	0	1	**19**	1	0.95
	**LR algorithm**						**GB algorithm**					
	EVOO/CO	EVOO/PO	EVOO/SBO	EVOO/SFO			EVOO/CO	EVOO/PO	EVOO/SBO	EVOO/SFO		
EVOO/CO	**19**	0	1	0	1	0.95	**12**	8	0	0	0.92	0.6
EVOO/PO	0	**20**	0	0	1	1	1	**19**	0	0	0.70	0.95

EVOO/SBO	0	0	**20**	0	0.91	1	0	0	**20**	0	0.95	1
EVOO/SFO	0	0	1	**19**	1	0.95	0	0	1	**19**	1	0.95
**Messenia**												
	**LDA algorithm**						**SVMs algorithm**					
	EVOO/CO	EVOO/PO	EVOO/SBO	EVOO/SFO			EVOO/CO	EVOO/PO	EVOO/SBO	EVOO/SFO		
EVOO/CO	**19**	1	0	0	1	0.95	**19**	1	0	0	1	0.95
EVOO/PO	0	**20**	0	0	0.95	1	0	**20**	0	0	0.95	1
EVOO/SBO	0	0	**20**	0	0.91	1	0	0	**19**	1	0.95	0.95
EVOO/SFO	0	0	2	**18**	1	0.9	0	0	1	**19**	0.95	0.95
	**LR algorithm**						**GB algorithm**					
	EVOO/CO	EVOO/PO	EVOO/SBO	EVOO/SFO			EVOO/CO	EVOO/PO	EVOO/SBO	EVOO/SFO		
EVOO/CO	**20**	0	0	0	1	1	**19**	1	0	0	1	0.95
EVOO/PO	0	**20**	0	0	1	1	0	**20**	0	0	0.87	1
EVOO/SBO	0	0	**19**	1	0.95	0.95	0	1	**19**	0	0.86	0.95
EVOO/SFO	0	0	1	**19**	0.95	0.95	0	1	3	**16**	1	0.8

Table S5. Confusion matrices and precision and recall scores for the different algorithms, using the fluorescence data, for each geographical region, considering one class for each adulterant (i.e., four classes in total).

**Fluorescence spectroscopy**												
**Achaia**												
	**LDA algorithm**						**SVMs algorithm**					
	**Predicted classes**						**Predicted classes**					
**Actual classes**	EVOO/CO	EVOO/PO	EVOO/SBO	EVOO/SFO	**Precision**	**Recall (Sensitivity)**	EVOO/CO	EVOO/PO	EVOO/SBO	EVOO/SFO	**Precision**	**Recall (Sensitivity)**
EVOO/CO	**20**	0	0	0	0.95	1	**20**	0	0	0	0.83	1
EVOO/PO	0	**20**	0	0	1	1	0	**20**	0	0	1	1
EVOO/SBO	0	0	**20**	0	1	1	0	0	**20**	0	1	1
EVOO/SFO	1	0	0	**19**	1	0.95	4	0	0	**16**	1	0.80
	**LR algorithm**						**GB algorithm**					
	EVOO/CO	EVOO/PO	EVOO/SBO	EVOO/SFO			EVOO/CO	EVOO/PO	EVOO/SBO	EVOO/SFO		
EVOO/CO	**20**	0	0	0	1	1	**20**	0	0	0	0.95	1
EVOO/PO	0	**17**	3	0	1	1	1	**19**	0	0	0.73	0.95
EVOO/SBO	0	0	**20**	0	1	1	0	7	**13**	0	1	0.65
EVOO/SFO	1	2	0	**17**	1	1	0	0	0	**20**	1	1
**Crete**												
	**LDA algorithm**						**SVMs algorithm**					
	EVOO/CO	EVOO/PO	EVOO/SBO	EVOO/SFO			EVOO/CO	EVOO/PO	EVOO/SBO	EVOO/SFO		
EVOO/CO	**20**	0	0	0	1	1	**20**	0	0	0	1	1
EVOO/PO	0	**20**	0	0	1	1	0	**20**	0	0	1	1
EVOO/SBO	0	0	**20**	0	1	1	0	0	**20**	0	1	1

EVOO/SFO	0	0	0	**20**	1	1	0	0	0	**20**	1	1
	**LR algorithm**						**GB algorithm**					
	EVOO/CO	EVOO/PO	EVOO/SBO	EVOO/SFO			EVOO/CO	EVOO/PO	EVOO/SBO	EVOO/SFO		
EVOO/CO	**20**	0	0	0	1	1	**20**	0	0	0	0.71	1
EVOO/PO	0	**20**	0	0	1	1	0	**20**	0	0	1	1
EVOO/SBO	0	0	**20**	0	1	1	8	0	**12**	0	0.75	0.60
EVOO/SFO	0	0	0	**20**	1	1	0	0	4	**16**	1	0.80
**Lesvos**												
	**LDA algorithm**						**SVMs algorithm**					
	EVOO/CO	EVOO/PO	EVOO/SBO	EVOO/SFO			EVOO/CO	EVOO/PO	EVOO/SBO	EVOO/SFO		
EVOO/CO	**20**	0	0	0	1	1	**20**	0	0	0	1	1
EVOO/PO	0	**20**	0	0	1	1	0	**20**	0	0	1	1
EVOO/SBO	0	0	**20**	0	0.95	1	0	0	**20**	0	1	1
EVOO/SFO	0	0	1	**19**	1	0.95	0	0	0	**20**	1	1
	**LR algorithm**						**GB algorithm**					
	EVOO/CO	EVOO/PO	EVOO/SBO	EVOO/SFO			EVOO/CO	EVOO/PO	EVOO/SBO	EVOO/SFO		
EVOO/CO	**19**	0	1	0	1	0.95	**12**	8	0	0	0.67	1
EVOO/PO	0	**20**	0	0	1	1	1	**19**	0	0	1	1
EVOO/SBO	0	0	**20**	0	0.95	1	0	0	**20**	0	1	1
EVOO/SFO	0	0	1	**19**	1	0.95	0	0	1	**19**	1	1
**Messenia**												
	**LDA algorithm**						**SVMs algorithm**					
	EVOO/CO	EVOO/PO	EVOO/SBO	EVOO/SFO			EVOO/CO	EVOO/PO	EVOO/SBO	EVOO/SFO		
EVOO/CO	**20**	0	0	0	1	1	**20**	0	0	0	1	1
EVOO/PO	0	**20**	0	0	1	1	0	**20**	0	0	1	1
EVOO/SBO	0	0	**20**	0	1	1	0	0	**20**	0	1	1
EVOO/SFO	0	0	0	**20**	1	1	0	0	0	**20**	1	1
	**LR algorithm**						**GB algorithm**					
	EVOO/CO	EVOO/PO	EVOO/SBO	EVOO/SFO			EVOO/CO	EVOO/PO	EVOO/SBO	EVOO/SFO		
EVOO/CO	**20**	0	0	0	1	1	**20**	0	0	0	0.91	1
EVOO/PO	0	**20**	0	0	1	1	0	**20**	0	0	0.71	1
EVOO/SBO	0	0	**20**	0	1	1	2	8	**10**	0	1	0.50
EVOO/SFO	0	0	0	**20**	1	1	0	0	0	**20**	1	1

Table S6. Confusion matrices and precision and recall scores for the different algorithms, using the LIBS, and the fluorescence data, for the geographical origin discrimination of pure EVOOs.

**LIBS**												
	**LDA algorithm**						**SVMs algorithm**					
	**Predicted classes**						**Predicted classes**					
**Actual classes**	Achaia	Crete	Lesvos	Messenia	**Precision**	**Recall (Sensitivity)**	Achaia	Crete	Lesvos	Messenia	**Precision**	**Recall (Sensitivity)**
Achaia	**20**	0	0	0	1	1	**20**	0	0	0	1	1
Crete	0	**20**	0	0	1	1	0	**19**	1	0	1	0.95
Lesvos	0	0	**20**	0	1	1	0	0	**20**	0	0.95	1
Messenia	0	0	0	**20**	1	1	0	0	0	**20**	1	1
	**LR algorithm**						**GB algorithm**					
	Achaia	Crete	Lesvos	Messenia			Achaia	Crete	Lesvos	Messenia		
Achaia	**20**	0	0	0	1	1	**20**	0	0	0	1	1
Crete	0	**19**	1	0	1	0.95	0	**18**	0	2	0.75	0.90
Lesvos	0	0	**20**	0	0.95	1	0	0	**20**	0	1	1
Messenia	0	0	0	**20**	1	1	0	6	0	**14**	0.88	0.70
**Fluorescence spectroscopy**												
	**LDA algorithm**						**SVMs algorithm**					
	Achaia	Crete	Lesvos	Messenia			Achaia	Crete	Lesvos	Messenia		
Achaia	**20**	0	0	0	0.77	1	**20**	0	0	0	0.56	1
Crete	0	**10**	10	0	1	0.50	10	**10**	0	0	1	0.50
Lesvos	0	0	**20**	0	0.67	1	0	0	**20**	0	1	1
Messenia	6	0	0	**14**	1	0.70	6	0	0	**14**	1	0.70
	**LR algorithm**						**GB algorithm**					
	Achaia	Crete	Lesvos	Messenia			Achaia	Crete	Lesvos	Messenia		
Achaia	**20**	0	0	0	0.95	1	**19**	0	0	1	1	0.95
Crete	1	**10**	9	0	1	0.50	0	**10**	10	0	0.50	0.50
Lesvos	0	0	**20**	0	0.69	1	0	10	**10**	0	0.33	0.50
Messenia	0	0	0	**20**	1	1	0	0	10	**10**	0.91	0.50

Table S7. Confusion matrices and precision and recall scores for the different algorithms, using the LIBS, and the fluorescence data, for the geographical origin discrimination of EVOO/non-EVOO mixtures.

	**LIBS**											
	**LDA algorithm**						**SVMs algorithm**					
	**Predicted classes**						**Predicted classes**					
**Actual classes**	Achaia	Crete	Lesvos	Messenia	**Precision**	**Recall (Sensitivity)**	Achaia	Crete	Lesvos	Messenia	**Precision**	**Recall (Sensitivity)**
Achaia	**80**	0	0	0	1	1	**80**	0	0	0	1	1
Crete	0	**79**	1	0	1	0.99	0	**80**	0	0	1	1
Lesvos	0	0	**79**	1	0.99	0.99	0	0	**79**	1	0.99	0.99
Messenia	0	0	0	**80**	0.99	1	0	0	1	**79**	099	0.99
	**LR algorithm**						**GB algorithm**					
	Achaia	Crete	Lesvos	Messenia			Achaia	Crete	Lesvos	Messenia		
Achaia	**80**	0	0	0	1	1	**77**	3	0	0	1	0.96
Crete	0	**78**	2	0	1	0.97	0	**78**	2	0	0.88	0.97
Lesvos	0	0	**79**	1	0.98	0.99	0	5	**72**	3	0.95	0.90
Messenia	0	0	0	**80**	0.99	1	0	3	2	**75**	0.96	0.94
	**Fluorescence spectroscopy**											
	**LDA algorithm**						**SVMs algorithm**					
	Achaia	Crete	Lesvos	Messenia			Achaia	Crete	Lesvos	Messenia		
Achaia	**80**	0	0	0	0.99	1	**79**	0	0	1	1	0.99
Crete	1	**79**	0	0	1	0.99	0	**80**	0	0	1	1
Lesvos	0	0	**80**	0	1	1	0	0	**80**	0	1	1
Messenia	0	0	0	**80**	1	1	0	0	0	**80**	0.99	**1**
	**LR algorithm**						**GB algorithm**					
	Achaia	Crete	Lesvos	Messenia			Achaia	Crete	Lesvos	Messenia		
Achaia	**80**	0	0	0	1	1	**78**	1	0	1	1	0.97
Crete	0	**80**	0	0	1	1	0	**80**	0	0	0.99	1
Lesvos	0	0	**80**	0	1	1	0	0	**80**	0	1	1
Messenia	0	0	0	**80**	1	1	0	0	0	**80**	0.99	1

Table S8. Confusion matrices and precision and recall scores for the different algorithms, using the LIBS, and the fluorescence data, for the geographical origin discrimination of EVOOs and their mixtures.

**LIBS**												
	**LDA algorithm**						**SVMs algorithm**					
	**Predicted classes**						**Predicted classes**					
**Actual classes**	Achaia	Crete	Lesvos	Messenia	**Precision**	**Recall (Sensitivity)**	Achaia	Crete	Lesvos	Messenia	**Precision**	**Recall (Sensitivity)**
Achaia	**100**	0	0	0	1	1	**100**	0	0	0	1	1
Crete	0	**99**	0	1	0.99	0.99	0	**100**	0	0	1	1
Lesvos	0	0	**100**	0	0.99	1	0	0	**100**	0	1	1
Messenia	0	1	1	**98**	0.99	0.98	0	0	0	**100**	1	1
	**LR algorithm**						**GB algorithm**					
	Achaia	Crete	Lesvos	Messenia			Achaia	Crete	Lesvos	Messenia		
Achaia	**100**	0	0	0	1	1	**94**	3	1	2	1	0.94
Crete	0	**99**	0	1	0.96	0.99	0	**96**	3	1	0.91	0.96
Lesvos	0	1	**98**	1	0.99	0.98	0	2	**98**	0	0.94	0.98
Messenia	0	3	1	**96**	0.98	0.96	0	4	2	**94**	0.97	0.94
**Fluorescence spectroscopy**												
	**LDA algorithm**						**SVMs algorithm**					
	Achaia	Crete	Lesvos	Messenia			Achaia	Crete	Lesvos	Messenia		
Achaia	**94**	5	0	1	0.90	0.94	**97**	0	0	3	1	0.97
Crete	4	**96**	0	0	0.94	0.96	0	**100**	0	0	0.97	1
Lesvos	0	0	**100**	0	0.98	1	0	0	**100**	0	1	1
Messenia	6	1	2	**91**	0.99	0.91	0	3	0	**97**	0.97	0.97
	**LR algorithm**						**GB algorithm**					
	Achaia	Crete	Lesvos	Messenia			Achaia	Crete	Lesvos	Messenia		
Achaia	**97**	2	0	1	0.94	0.97	**77**	15	1	8	0.99	0.77
Crete	3	**97**	0	0	0.96	0.97	1	**82**	0	17	0.83	0.82
Lesvos	0	0	**100**	0	1	1	0	2	**96**	2	0.91	0.96
Messenia	3	2	0	**95**	0.99	0.95	0	0	10	**90**	0.77	0.90
